# Global Gene Expression Analysis Reveals Dynamic and Developmental Stage–Dependent Enrichment of Lead-Induced Neurological Gene Alterations

**DOI:** 10.1289/ehp.1002590

**Published:** 2010-12-08

**Authors:** Samuel M. Peterson, Jun Zhang, Gregory Weber, Jennifer L. Freeman

**Affiliations:** School of Health Sciences, Purdue University, West Lafayette, Indiana, USA

**Keywords:** Danio rerio, gene expression, genomics, lead, microarray, Pb, toxicogenomics, zebrafish

## Abstract

**Background:**

The underlying genetic mechanisms specific to subtle neurological alterations associated with environmental lead (Pb) exposures have not been clearly elucidated.

**Objectives:**

The goal of this study was to identify novel gene targets and the underlying genetic mechanisms associated with developmental Pb neurotoxicity.

**Methods:**

We first exposed zebrafish embryos to a range of Pb concentrations throughout early development to establish relative toxicity. Using the data from that experiment, we exposed another group of zebrafish embryos to a sublethal dose of Pb (100 ppb) immediately after fertilization through 72 or 120 hr postfertilization (hpf). Global gene expression was then analyzed for molecular pathways and gene ontology enrichment, and Western blot analysis was performed to investigate the translation of gene expression changes to protein levels.

**Results:**

After 72 hpf, we identified 231 probes representing 90 nonredundant genes with well-established function or orthology to human genes as being altered by Pb exposure. This gene set was both confirmatory and novel in nature and was highly enriched for neurological development, function, and disease. Moreover, gene changes at this time point were correlated to altered protein levels. Alternatively, the gene set at 120 hpf did not share association with neurological development.

**Conclusions:**

Global gene expression alterations associated with developmental Pb exposure were dynamic and dependent on developmental stage. Gene expression alterations at the 72-hpf time point were highly enriched with genes and molecular pathways associated with neurological development and disease. Moreover, we identified a number of novel targets for future exploration into their role in the genetic mechanisms underlying Pb-induced neurological alterations.

The heavy metal lead (Pb) is a well-studied toxicant known to cause a wide array of adverse health effects. The developing nervous system is especially susceptible to Pb-induced alterations, and the Centers for Disease Control and Prevention (CDC) currently recommends that primary prevention strategies be implemented when a child’s blood Pb level (BLL) exceeds 10 μg/dL (CDC 2005). However, increasingly stringent regulatory actions have failed to limit exposures to a level documented as safe and without neurological impacts (Canfield et al. 2003; Min et al. 2007), including recent epidemiological studies that linked subinterventional BLLs to decreased IQ (intelligence quotient) and attention span and increased behavioral problems and odds ratio for the development of attention deficit hyperactivity disorder (Eubig et al. 2010, Surkan et al. 2007). Moreover, although the mechanisms of Pb toxicity have been studied for many years, the molecular mechanisms underlying the subtle neurological impacts are not understood.

The developing nervous system is particularly susceptible to Pb toxicity because of increased levels of cell proliferation, migration, and differentiation and the complexity of cell interactions (Rodier 1994, 2004). In addition, children absorb a greater percentage of ingested Pb, and circulating Pb has greater access to the brain because the blood–brain barrier is not yet fully established (Cory-Slechta and Schaumburg 2000; Leggett 1993). Also, Pb can be released from maternal bone stores and transferred to a developing fetus (Gardella 2001). As a result, Pb exposure can be initiated early in prenatal development, and studies have indicated that umbilical BLLs ≤ 10 μg/dL are linked with deleterious effects on cognitive development (Gomaa et al. 2002). Thus, it is important to study Pb exposure *in vivo* during embryogenesis at a relevant concentration.

The zebrafish presents a complementary model to rodents to assess impacts of prenatal chemical exposure. To specifically assess adverse effects associated with chemical exposure during embryonic development in rodents, dam dosing and sacrifice are generally required. Alternatively, the zebrafish model has gained popularity in developmental and toxicological studies because of rapid *ex utero* embryonic development in which embryos can be dosed in a petri dish. Additionally, a near-transparent chorion permits easy visualization of the developing organism throughout the entire embryonic period. Moreover, a single breeding pair can produce hundreds of embryos weekly, providing a large pool of individuals for experimental testing. The application of the zebrafish model in developmental biology research has resulted in a plethora of background literature, as well as the incorporation of the species into genetic and toxicology research. Moreover, a finished genome sequence and the homology between zebrafish and human genes permit translation of molecular mechanisms of toxicity observed in the zebrafish model system to humans (Barbazuk et al. 2000). Thus, the zebrafish provides an ideal model organism for defining molecular alterations associated with early developmental Pb exposure.

Although numerous studies have investigated Pb toxicity (see “Discussion” for examples), the molecular mechanisms underlying the subtle neurological effects on the developing nervous system are not thoroughly understood. The use of microarray technology allows for the simultaneous measurement of the expression level of genes on a genomewide scale. When used with proper statistical approaches and genetic pathway analyses, a list of statistically significant, differentially expressed genes can be combined with an extensive database of gene and gene product interactions to provide a robust determination of commonly affected gene networks and pathways (Mattingly et al. 2009; Yang et al. 2007). Based on the results of these calculations, the ability to predict and make hypotheses about specific mechanisms of toxicity is strongly improved. In this study we aimed to identify key genes and regulatory networks perturbed by sublethal developmental Pb exposure. Our goal was to achieve enrichment of genes and molecular pathways associated with neurological development and disease in order to identify novel gene targets for future investigation into the specific genetic mechanisms underlying developmental Pb toxicity at sublethal doses. Using zebrafish *ex utero* development, we initiated exposure conditions immediately after fertilization to correspond to the mammalian *in utero* prenatal developmental period. We combined genomewide expression analysis with pathway analysis to uncover novel genes of interest and to highlight critical time points in development. Further, we investigated translation of gene expression alterations to the protein level.

## Materials and Methods

### Zebrafish husbandry

Zebrafish used in this study were of the wild-type AB strain; they were housed on a 14:10-hr light:dark cycle in a Z-Mod System (Aquatic Habitats, Apopka, FL) at 28°C. Adults were bred in spawning cages according to established protocols to obtain staged embryos (Westerfield 2000). For Pb treatment, embryos were collected at the 2- to 16-cell stage, washed, and sorted for experimental procedures. All protocols were approved by Purdue University’s Institutional Animal Care and Use Committee, with all fish treated humanely and with regard to alleviation of suffering.

### Relative toxicity of Pb in zebrafish

We first performed an acute toxicity test to establish and confirm the relative toxicity of Pb to zebrafish and to determine an appropriate dose for gene expression analysis. Fifty embryos (considered as subsamples) were exposed to either control conditions (filtered aquarium water) or Pb treatment (Pb acetate; Sigma, St. Louis, MO) immediately after fertilization. Pb concentrations ranged from 1 to 100,000 ppb (micrograms per liter). Embryos were monitored at 6-hr intervals for mortality, hatching time, and developmental malformations through 120 hr postfertilization (hpf). We conducted three replicate experiments (*n* = 3) and performed analysis of variance (ANOVA) with SAS software (version 9.2; SAS Institute Inc., Cary, NC) by developmental time point. Means were compared using the least significant difference test at α = 0.05 when a significant ANOVA was observed.

### Global gene expression analysis

Based on the relative toxicity assessment, we selected 100 ppb Pb as the experimental treatment for gene expression analysis. This experimental treatment represents a concentration that is well below the tested concentrations at which zebrafish exhibited mortality, alterations in hatching time, and gross morphological malformations. Fifty embryos (considered as subsamples) were exposed to either control conditions or 100 ppb Pb as described above through 72 or 120 hpf. These two time points correlate, respectively, with *a*) the end of the embryonic phase and *b*) the initiation of complex behaviors such as survival and hunting at which formation of the major brain structures is complete (Westerfield 2000). At the end of the treatment period, embryos were pooled, homogenized in Trizol (Invitrogen, Carlsbad, CA), and flash frozen in liquid nitrogen. Samples were stored at −80°C until RNA isolation was performed. Three biological replicates were completed (*n* = 3) at both time points. Water samples were collected and analyzed for Pb concentration at an inductively coupled plasma mass spectroscopy core facility, where measurement accuracy and solubility were confirmed.

Total RNA was isolated from embryos and converted to cDNA following established protocols (Peterson and Freeman 2009b). Microarray analysis was performed according to Peterson and Freeman (2009a), with labeled samples hybridized onto the zebrafish 385K expression microarray platform (Roche NimbleGen, Madison, WI) designed by Y. Zhou and L. Zon (Harvard Medical School and Children’s Hospital Boston, Boston, MA) using the one-color hybridization strategy. This platform contains 385,000 60-mer probes interrogating 37,157 targets with up to 12 probes per target. After hybridization, arrays were washed and scanned at 5 μm with a GenePix 4000B array scanner (Molecular Devices, Sunnyvale, CA).

Array image data were extracted using the NimbleScan software program (Roche NimbleGen). Fluorescence signal intensities were normalized using quantile normalization (Bolstad et al. 2003), and gene calls were generated using the Robust Multichip Average algorithm (Irizarry et al. 2003) following manufacturer recommendations.

### Statistical processing

Further statistical processing of array data was performed with Array Star (version 3.0; DNASTAR, Inc., Madison, WI) and Ingenuity Pathway Analysis (IPA) software (version 8.0; Ingenuity Systems, Redwood City, CA) using a multitiered approach to determine a robust and reproducible list of differentially expressed genes and to identify gene functions, pathways, and ontology groups enriched by this list. Criteria used to classify genes as being differentially expressed were adapted from recent recommendations from the Microarray Quality Control Consortium (Guo et al. 2006; Shi et al. 2006). Two criteria were used to generate a list of significantly and consistently altered genes: *a*) genes consistently expressed (Student’s *t*-test, *p* ≤ 0.1) and substantially altered at a mean absolute log_2_ expression ratio of at least 0.585 (50% increase or decrease in expression); and *b*) genes consistently altered by a minimum absolute log_2_ expression ratio of 0.585 in all three replicates. Differently expressed gene sets were imported into IPA to establish gene classifications and gene enrichment categories.

### Quantitative polymerase chain reaction (qPCR) comparison

We compared microarray data with qPCR on a set of selected genes. Probes specific for these genes were designed using the Primer3 website (SourceForge 2010) as described by Rozen and Skaletsky (2000) [see Supplemental Material, Table 1 (doi:10.1289/ehp.1002590)]. qPCR analysis was performed on a Stratagene MX3000P qPCR system (Agilent, La Jolla, CA) using iQ SYBR Green Supermix kit (Bio-Rad, Hercules, CA) according to the manufacturer’s recommendations. The cycling parameters included a 3-min incubation phase at 95°C, 40 cycles of 95°C for 10 sec, 60°C for 30 sec, and 72°C for 30 sec, and a dissociation curve to check for nonspecific amplification. Efficiency and specificity were checked with melting and dilution curve analysis and no-template controls. Threshold values were calculated, and individual gene expression was normalized to glyceraldehyde-3-phosphate dehydrogenase (*GAPDH*).

### Protein level analysis

We performed Western blot analysis to determine if gene expression alterations were correlated to protein levels of altered genes. Protein was isolated from whole embryos exposed to control treatment or to 100 ppb Pb using Cell Lytic MT Mammalian Tissue Lysis/Reagent (Sigma). Protein isolates were separated using sodium dodecyl sulfate–polyacrylamide gel electrophoresis and transferred to a polyvinyl difluoride membrane (Bio-Rad). We obtained primary antibodies for metallothionein 2 (MT2; ab12228), FRY-like (FRYL; ab95065), and GAPDH (ab8245) from Abcam (Cambridge, MA); for reelin (RELN; anti-RELN clone 142) from Millipore (Billerica, MA); and for mitogen-activated protein kinase 8 (MAPK8; anti-MAPK-8 CT) from AnaSpec (Fremont, CA). After incubation with appropriate horseradish peroxidase–conjugated secondary antibodies, protein bands were visualized with the Immun-Star WesternC Chemiluminescence Kit on a ChemiDoc XRS+ imager (both from Bio-Rad). We used Image Lab Software (Bio-Rad) to calculate relative quantification of three biological replicates after normalizing proteins of interest to GAPDH. An unpaired Student’s *t*-test was used to determine significant alterations in protein levels.

## Results

### Relative toxicity of Pb in zebrafish

**Figure 1:**
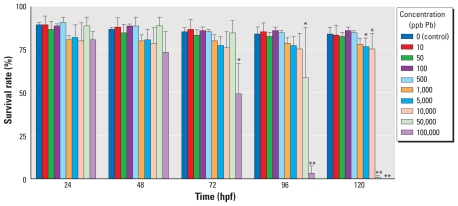
Acute toxicity test for mortality in zebrafish embryos exposed to a range of Pb concentrations for 120 hpf and monitored for severe malformations and death. The survival rate is presented as the percentage (mean ± SD) of embryos surviving at each time point, determined using three replicates of 50 embryos each. *p ≤ 0.05, and **p ≤ 0.0001 compared with controls.

We performed an acute toxicity test through 120 hpf to characterize the general response of zebrafish embryos to developmental Pb exposure, with specific end points of survival and hatching rate. We observed a dose response in mortality rates, with higher concentrations displaying greater levels of embryo mortality and with deaths occurring closer to the onset of the experiment (Figure 1). Embryos treated with the highest Pb concentrations (100,000 and 50,000 ppb) had a survival percentage that began to differ from control (*p* ≤ 0.05) at 54 and 96 hpf, respectively, and reached near or complete mortality before the end of the experiment. All Pb concentrations ≥ 5,000 ppb reached a level of significant difference in mortality by 120 hpf (*p* ≤ 0.05; Figure 1).

**Figure 2:**
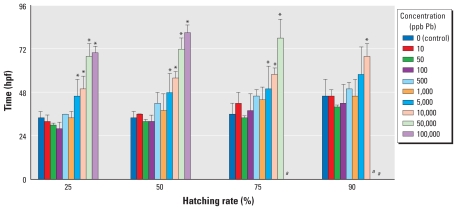
Acute toxicity test for hatching rate (percentage of surviving embryos hatched) alterations in zebrafish embryos after exposure to various concentrations of Pb. Values represent (mean ± SD) time required (hpf) to reach levels of hatching (*n* = 3, with 50 subsamples per replicate). *^a^*Data were excluded because analysis of this end point was confounded by concurrent mortality rates in the treatment group. *p ≤ 0.05 compared with controls.

To obtain an estimate of hatching delay, we compared the average time it took treatment groups to obtain a specific level of hatching (Figure 2). We excluded data from the analysis when levels of increased mortality interfered with interpretation (i.e., when mortality rates were different from controls at time points reported above). Embryos treated with all Pb concentrations ≥ 5,000 ppb demonstrated a significant delay in hatching compared with controls (*p* ≤ 0.05). Control embryos obtained a 50% hatching rate by a mean time of 34 hpf, whereas the embryos treated with 5,000 and 10,000 ppb Pb were delayed and did not reach a 50% hatching rate until the mean times of 48 and 56 hpf, respectively. We observed more severe delays in the highest two concentrations, 50,000 ppb and 100,000 ppb, with mean hatching times of 72 and 81 hpf, respectively.

### Global gene expression analysis at 72 hpf

**Figure 3:**
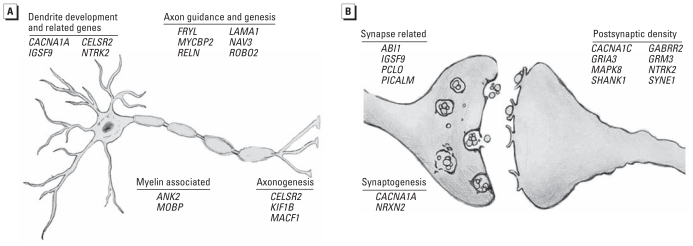
Schematic representation of altered neurological gene enrichment at 72 hpf after exposure to 100 ppb Pb, enriched with genes related to neurological function. All gene ontology categories were gathered from the Database for Annotation, Visualization and Integrated Discovery (DAVID) Bioinformatics Resources 6.7 (Huang et al. 2009a, 2009b), with the exception of *FRYL* and *NAV3*, which were categorized from Emoto et al. (2004) and Maes et al. (2002), respectively. Among the set of 90 altered genes, enrichment was seen in genes relating to neurite projections (*A*) and synapse function (*B*). See Table 2 for full gene names.

From the results of the relative toxicity assay, we chose an exposure concentration of 100 ppb for global gene expression analysis to represent a concentration well below one that induced overt toxicity. Global gene expression analysis performed on zebrafish embryos at 72 hpf resulted in a scatter plot with an *R*^2^ correlation of 0.9813 [see Supplemental Material, Figure 1A (doi:10.1289/ehp.1002590)]. We observed a total of 231 altered probes at this time point (see Supplemental Material, Table 2). After accounting for redundant probes that targeted identical genes and removing probes targeting hypothetical proteins without substantial function or ontology information, significant annotation and network information was available for 90 genes orthologous to human genes with established functions. Gene ontology analysis performed with IPA software classified genes into nonexclusive categories (i.e., genes can belong to more than one related or distinct category). This list of genes was highly enriched for genetic disorders and neurological disease, as well as several molecular functions and physiological systems (Table 1). Specifically, a number of genes were associated with one or more genetic or neurological disorders, including Alzheimer’s disease, non-insulin-dependent diabetes, bipolar disorder, and Parkinson’s disease (Table 1). We observed significant enrichment with 55 of the 90 genes involved in neurological development, function, and disease with specific areas affected, including synaptic transmission, long-term potentiation (LTP), guidance of axons, and branching of neurites (Table 2, Figure 3A,B). Overall, this gene list included targets that were both confirmatory, representing genes previously identified to be associated with Pb toxicity, and novel in nature.

### Global gene expression analysis at 120 hpf

The expression patterns of Pb-treated and control embryos differed less from each other at 120 hpf. The *R*^2^ correlation of the resulting scatter plot was 0.9918, with 82 probes being classified as significantly altered [see Supplemental Material, Figure 1B, Table 3 (doi:10.1289/ehp.1002590)]. Of these 82 probes, significant annotation and functional information was available for 30 nonredundant genes. The gene list for this developmental time point did not share the same association as the 72 hpf set and displayed some association with renal, urological, and hematological disorders, as well as cancer, lipid metabolism, tissue development, and cardiovascular system development and function (see Supplemental Material, Table 4).

### Comparison of microarray and qPCR data

We compared microarray data with qPCR on a subset of genes. Probes selected comprised genes altered at 72 hpf or at 120 hpf, with most genes relating to neurological development [see Supplemental Material, Table 1 (doi:10.1289/ehp.1002590)]. We observed a high level of agreement between the genes determined to be altered according to microarray data and the qPCR analysis (assessed by two-sided *t*-test, *p* ≤ 0.05).

### Protein level analysis

**Figure 4:**
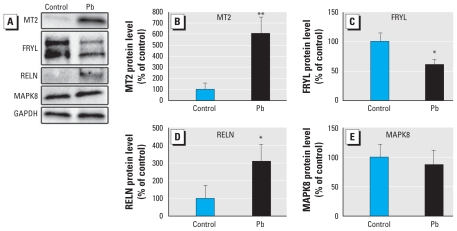
Protein levels of four genes with altered expression at 72 hpf (*MT2, FRYL, RELN,* and *MAPK8*) as determined by Western blot analysis (*A*). Differences in protein levels of genes of interest in zebrafish embryos following exposure to 100 ppb Pb were compared with controls after being normalized to GAPDH (*B*). All blots are representative of results of three biological replicates. Protein levels of MT2 (*B*), FRYL (*C*), and RELN (*D*) were all altered in the Pb-treated embryos, whereas MAPK8 (*E*) did not demonstrate a significant alteration. *p ≤ 0.05, and **p ≤ 0.01.

Using Western blot analysis, we measured relative changes in the protein levels of MT2, FRYL, RELN, and MAPK8. Significant alterations were present in MT2, RELN, and FRYL levels in Pb-treated samples, whereas MAPK8 appeared to be unchanged (Figure 4). The levels of MT2 protein increased to > 600% of control (*p* = 0.006). The antibody for RELN recognized multiple bands that correspond to the full-length protein as well as expected processed fragments. The levels of the unprocessed form were increased to 309% of control in the treated samples (*p* = 0.0425). FRYL protein was markedly decreased, as evidenced by two bands representing products of similar size (larger product: 63% of control, *p* = 0.047; smaller product: 57% of control, *p* = 0.016; combined: 60.2% of control, *p* = 0.020). MAPK8 protein levels did not significantly change with Pb treatment (*p* = 0.53).

## Discussion

In the present study, we characterized gene expression alterations induced by Pb exposure at two early developmental time points in order to provide a framework for assessing the biological pathways and functions that are perturbed and susceptible to a sublethal dose of Pb. Based on established research and epidemiological studies, we hypothesized that the developing nervous system would be the most sensitive to Pb-induced alterations and that genes relating to these processes would be among the first to be altered. At higher Pb doses, pathways that are altered by lower doses of Pb are likely to be indistinguishable in the multitude of drastic gene alterations that accompany more general toxic responses (e.g., oxidative stress, heat-shock protein–associated expression, apoptosis). Furthermore, exposures to high doses of Pb can result in myriad effects, damaging most organ systems. The interpretation of neurologically based effects would be compromised by using a dose in which other effects are prominently observed.

The complex binding dynamics and absorption proprieties of Pb do not permit easy approximation of the zebrafish developmental dose equivalent to a child with a BLL in the range of interest (1–10 μg/dL, equivalent to 10–100 ppb). To accomplish this end and to approximate a relevant dose, we first performed a relative toxicity assay to characterize the general response of the embryos to a range of Pb concentrations. Each group of embryos treated with concentrations ≥ 5,000 ppb demonstrated a significant delay in hatching and increased mortality. The highest concentrations elicited almost complete mortality and interfered with analysis of hatching rates at later developmental time points. As a result of these data, we chose an exposure concentration of 100 ppb (0.48 μM, equivalent to 10 μg/dL). Embryos treated at this concentration were consistently similar to controls at all developmental time points analyzed and did not elicit any observable toxicity.

The use of *in vivo* and *in vitro* models has demonstrated a number of structural and functional neurotoxic consequences of Pb exposure and provides support for data from epidemiological studies (Min et al. 2007). However, the molecular mechanisms underlying the subtle neurological alterations associated with environmental Pb exposure during development are not completely understood. In the present study, we used microarray technology to identify gene targets and unravel the underlying molecular mechanisms of neurodevelopmental alterations associated with sublethal Pb exposure in an *in vivo* system during early development. The molecular targets altered at 72 hpf included genes that were both confirmatory and novel in nature. Confirmatory genes previously reported as having altered expression or gene product function after Pb exposure and that are involved in neurological development, function, or disease included *LRP1* (low density lipoprotein receptor-related protein 1) (Behl et al. 2009), *MAPK8* (Posser et al. 2007), *CSF1R* (colony stimulating factor 1 receptor) (Kowolenko et al. 1989), *GRM3* (glutamate receptor, metabotropic 3) (Xu et al. 2007), *MT2A* (metallothionein 2A) (Wang et al. 2009), and *POU2F1* (POU class 2 homeobox 1) (Glahn et al. 2009). Moreover, the list of genes altered at 72 hpf related not only to general nervous system development but specifically to distinct functions previously reported as perturbed by Pb exposure in which established specific molecular targets are lacking. These novel gene targets provide additional insight into the specific molecular mechanisms associated with established Pb-induced functional alterations, including genes related to neuronal ontogenesis and synapse formation and function.

Neuronal ontogenesis has been reported to be perturbed by Pb exposure through a reduction in neuron growth, axon and dendrite branching, and altered expression of neural cell adhesion molecules (N-CAMs; Figure 3A) (Cory-Slechta and Schaumburg 2000). The most notable of the altered genes in this experiment that are associated with these functions include *RELN*, a gene with multiple functions throughout early nervous system development, including a role in activating a signal cascade for regulating neuronal migration (Jossin 2004), and *ROBO2* [roundabout, axon guidance receptor, homolog 2 (*Drosophila*)], a gene that activates a repulsive guidance signal for neurons (López-Bendito et al. 2007). Additional genes of note that have been implicated in the growth and guidance of axons and that we observed to have altered expression at 72 hpf include *FRYL*, *LAMA1* (laminin, alpha 1), *MYCBP2* (*MYC* binding protein 2), and *NAV3* (neuron navigator 3). Furthermore, the altered behavior and expression of N-CAMs are associated with Pb exposure. The disruption of these glycoproteins has been implicated as a contributor to the altered brain morphogenesis observed in animal models, because the N-CAMs play a key role in cell–cell interactions such as neurite outgrowth and regulation of synaptic contacts (Shi et al. 2004). Specifically, *IGSF9* (immunoglobulin superfamily, member 9), a member of the N-CAM family, demonstrated altered expression at 72 hpf in the Pb-exposed embryos in the present study.

Synapse formation and the regulation of LTP are established phenotypic consequences of Pb exposure, and a large number of genes linked to these processes were altered at 72 hpf (Figure 3B) (Gilbert et al. 1999). These targets included genes for multiple neurotransmitter receptors (*GRIA3*, *GRM3*, and *GABRR2*), *PCLO* [piccolo (presynaptic cytomatrix protein)], and *NTRK2* (neurotrophic tyrosine kinase, receptor, type 2). Pb has been shown to interfere with both the glutamate and GABAergic neurotransmitter systems that play important roles in maintaining synaptic plasticity and the formation of LTP (Braga et al. 1999). Further association with genes relating to LTP can be seen with the alterations in gene expression observed in *PCLO*, *NTRK2*, and the previously discussed *IGSF9* and *RELN*. Overall, multiple genes altered after Pb exposure in this study are associated with functions previously indicated to be perturbed by Pb exposure but for which molecular targets are currently lacking.

To investigate whether alterations of gene expression identified in the present study translate to the protein level, we performed Western blot analysis to measure the relative protein levels of four molecular targets with altered gene expression: *MT2*, *RELN*, *FRYL*, and *MAPK8*. The most striking result came from the dramatic increase in MT2 levels. The increase in the full-length RELN protein is also interesting in that levels of this unprocessed form are generally extremely low *in vivo*, and processed forms are predominantly observed (Tissir and Goffinet 2003). The reason for the accumulation of unprocessed protein is not known, but we hypothesize it to be a result of Pb interfering with proper protein cleavage. The FRYL protein products we detected in the present study are shorter than the human protein, but this protein has yet to be characterized in the zebrafish. Regardless, we observed a decrease in the relative amounts of FRYL in the Pb-treated embryos compared with controls. Further research is needed to determine the exact structure and functional role of these products. Overall, we observed altered protein levels for three of the four molecular targets, indicating that gene expression alterations are being translated to the protein level.

Interestingly, at the later time point analyzed (120 hpf), we did not observe an association with neurological development, and overall, fewer genes had altered expression. One gene of interest, *POMC* (proopiomelanocortin), has previously been associated with low-level Pb exposure (Rosen and Polakiewicz 1989). In addition, we observed no overlap between genes with altered expression at the two developmental time points. Of note, by 120 hpf, many of the zebrafish organ systems, including the nervous system, have progressed through much of organogenesis. The high degree of rapidly dividing, differentiating, and migrating cells involved in the key stages of brain development have largely stabilized by 120 hpf (Mueller and Wullimann 2005). It is likely that these processes account for the high degree of Pb-induced susceptibility responsible for the alterations observed at 72 hpf, supporting the notion that toxicogenomic responses are developmental stage–dependent and represent a snapshot of the dynamic developmental processes occurring at specific time points.

Overall, in the present study we *a*) identified a number of novel genetic targets associated with developmental exposure to a sublethal Pb concentration, *b*) identified enrichment of genes associated specifically with neurite growth and inhibition of LTP and synapse formation at 72 hpf, and *c*) found that gene expression changes are being translated to the protein level. Alternatively, we did not observe an association between perturbed genes and neurological function at 120 hpf. We found a lack of overlap between the neurological genes altered in our study compared with other genomic studies of Pb toxicity (Bouton et al. 2001; Yang et al. 2007). Differences may be attributed to dissimilarity in experimental design, including experimental systems, dose, and specific developmental time points analyzed. Although at the present time it is unclear whether the altered gene expression resulted in permanent alterations, the gene targets and molecular pathways identified at 72 hpf support a molecular basis for alterations of neurite growth and synapse formation and function that have been observed in previous studies. Future studies will focus on further elucidating and correlating the molecular basis of phenotypic alterations associated with sublethal doses of Pb exposure.

## Conclusion

We exposed zebrafish embryos to a sublethal dose of Pb and analyzed global transcriptional alterations at two developmental time points. Only during the earlier time point were genes relating to neurodevelopment and function selectively altered. The differing results acquired at the two developmental time points highlight the importance of careful assessment of exposure duration and of specific time points in developmental toxicology studies, because gene expression is dynamic throughout development and represents a snapshot of the molecular processes representative of that developmental stage.

## Figures and Tables

**Table 1:** Gene ontology of altered genes at 72 hpf.

Function category	*p*-Value^a^	No. of genes^b^
Genetic disorder or neurological disease		
Alzheimer’s disease	3.05E–05	16
Non-insulin-dependent diabetes	6.19E–05	20
Progressive motor neuropathy	1.45E–02	13
Coronary artery disease	2.34E–03	15
Neurodegenerative disorder	1.64E–05	17
Parkinson’s disease	6.28E–03	9

Cellular development		
Developmental processes of axons	4.78E–02	5
Shape change of neurons	6.81E–03	2

Cell-to-cell signaling interaction		
Long-term depression of cells	6.34E–04	5
Synaptic transmission	8.60E–04	7

Cellular assembly and organization		
Density of synaptic vesicles	1.58E–04	2
Quantity of synaptic vesicles	9.34E–04	2
Growth of neurites	3.11E–03	7
Morphogenesis of neurites	3.60E–03	4

Cell morphology		
Morphogenesis of dendrites	4.08E–03	3
Branching of neurites	5.73E–03	3
Depolarization	4.27E–03	3

Nervous system development and function		
Neurological processes of axons	1.12E–03	5
Guidance of axons	2.55E–02	2
LTP	6.34E–04	5

LTP, long-term potentiation.

a Derived from the likelihood of observing the degree of enrichment in a gene set of a given size by chance alone.

b Classified as being differentially expressed that relate to the specified function category; a gene may be present in more than one category.

**Table 2:** Neurological genes altered at 72 hpf.

Symbol	Gene name^a^	Log_2_ expression ratio
*ABI1*	*abl*-interactor 1	−0.84
*ANK2*	ankyrin 2, neuronal	−0.67
*ANO3*	anoctamin 3	−0.90
*AP3D1*	adaptor-related protein complex 3, delta 1 subunit	−0.97
*BAT2*	*HLA-B* associated transcript 2	−1.74
*CACNA1A*	calcium channel, voltage-dependent, P/Q type, alpha 1A subunit	−1.05
*CACNA1C*	calcium channel, voltage-dependent, L type, alpha 1C subunit	−1.18
*CELSR3*	cadherin, EGF LAG seven-pass G-type receptor 3 (flamingo homolog, Drosophila)	−1.45
*COL11A2*	collagen, type XI, alpha 2	−1.99
*CSF1R*	colony stimulating factor 1 receptor	0.90
*CTSL2*	cathepsin L2	0.61
*ENPP5*	ectonucleotide pyrophosphatase/phosphodiesterase 5	−0.74
*ERAP1*	endoplasmic reticulum aminopeptidase 1	0.59
*FAT1*	*FAT* tumor suppressor homolog 1 (Drosophila)	−1.00
*FRYL*	*FRY*-like	−1.62
*GABRR2*	gamma-aminobutyric acid (GABA) receptor, rho 2	0.66
*GRIA3*	glutamate receptor, ionotrophic, AMPA 3	−0.75
*GRM3*	glutamate receptor, metabotropic 3	−0.70
*HEATR5B*	HEAT repeat containing 5B	−0.91
*HIVEP2*	human immunodeficiency virus type I enhancer binding protein 2	−0.84
*IGSF9*	immunoglobulin superfamily, member 9	−0.69
*KCNH8*	potassium voltage-gated channel, subfamily H (eag-related), member 8	−0.72
*KIF1A*	kinesin family member 1A	−0.99
*KIF1B*	kinesin family member 1B	−1.17
*LAMA1*	laminin, alpha 1	−0.81
*LRP1*	low density lipoprotein receptor-related protein 1	−1.54
*LRP1B*	low density lipoprotein receptor-related protein 1B	−0.77
*MACF1*	microtubule-actin crosslinking factor 1	−1.21
*MADD*	MAP-kinase activating death domain	−0.89
*MAPK8*	mitogen-activated protein kinase 8	−0.87
*MBD5*	methyl-CpG binding domain protein 5	−1.04
*MLL3*	myeloid/lymphoid or mixed-lineage leukemia 3	−0.69
*MOBP*	myelin-associated oligodendrocyte basic protein	−0.66
*MT2A*	metallothionein 2A	0.87
*MYCBP2*	*MYC* binding protein 2	−1.06
*NAV3*	neuron navigator 3	−0.77
*NCOR2*	nuclear receptor co-repressor 2	−1.21
*NRXN2*	neurexin 2	−1.29
*NTRK2*	neurotrophic tyrosine kinase, receptor, type 2	−0.80
*OSBPL6*	oxysterol binding protein-like 6	−0.62
*PCLO*	piccolo (presynaptic cytomatrix protein)	−1.01
*PDE4DIP*	phosphodiesterase 4D interacting protein	−1.94
*PICALM*	phosphatidylinositol binding clathrin assembly protein	−0.78
*POU2F1*	POU class 2 homeobox 1	−0.78
*RELN*	reelin	−1.32
*ROBO2*	roundabout, axon guidance receptor, homolog 2 (Drosophila)	−1.36
*RYR3*	ryanodine receptor 3	−1.34
*SHANK1*	SH3 and multiple ankyrin repeat domains	−0.94
*SNX26*	sorting nexin 26	−1.02
*SREBF1*	sterol regulatory element binding transcription factor 1	−0.97
*SYNE1*	spectrin repeat containing, nuclear envelope 1	−0.67
*UBR4*	ubiquitin protein ligase E3 component n-recognin 4	−1.31
*VPS13B*	vacuolar protein sorting 13 homolog B (yeast)	−0.61
*WDFY3*	WD repeat and FYVE domain containing 3	−1.22
*WDR7*	WD repeat domain 7	−1.09

a From National Center for Biotechnology Information (2010).
